# Supplementary material for: COZOID: contact zone identifier for visual analysis of protein-protein interactions
Source: BMC Bioinformatics. 2018 Apr 6;19:125. doi: 10.1186/s12859-018-2113-6 (PMC5889581; doi:10.1186/s12859-018-2113-6)
Supplement: Supplementary file 2 — Software build. Executable binary file of the software tool. (ZIP 118,784 kb) [file 12859_2018_2113_MOESM2_ESM.zip › cozoid/java/docs/beansbinding-1.2.1-doc.zip!/index-files/index-13.html]

M-Index (Beans Binding)


---


|  |  |  |
| --- | --- | --- |
| **Overview** \| Package \| Class \| Use \| **Tree** \| **Deprecated** \| **Index** \| **Help** | |  |
| **PREV LETTER**   **NEXT LETTER** | **FRAMES**    **NO FRAMES**     **All Classes** |  |


A B C D E F G H I J K L M N O P Q R S T U V W 

---


## **M**

**m** - Variable in class org.jdesktop.el.impl.lang.FunctionMapperImpl.Function: **MapELResolver** - Class in org.jdesktop.el: Defines property resolution behavior on instances of `Map`. **MapELResolver()** - Constructor for class org.jdesktop.el.MapELResolver: Creates a new read/write `MapELResolver`. **MapELResolver(boolean)** - Constructor for class org.jdesktop.el.MapELResolver: Creates a new `MapELResolver` whose read-only status is determined by the given parameter. **mapKeyAdded(ObservableMap, Object)** - Method in interface org.jdesktop.observablecollections.ObservableMapListener: Notification that a key has been added. **mapKeyRemoved(ObservableMap, Object, Object)** - Method in interface org.jdesktop.observablecollections.ObservableMapListener: Notification that a key has been removed **mapKeyValueChanged(ObservableMap, Object, Object)** - Method in interface org.jdesktop.observablecollections.ObservableMapListener: Notification that the value of an existing key has changed. **matches(Object, Object)** - Method in class org.jdesktop.el.impl.lang.ELArithmetic.BigDecimalDelegate: **matches(Object, Object)** - Method in class org.jdesktop.el.impl.lang.ELArithmetic.BigIntegerDelegate: **matches(Object, Object)** - Method in class org.jdesktop.el.impl.lang.ELArithmetic.DoubleDelegate: **matches(Object, Object)** - Method in class org.jdesktop.el.impl.lang.ELArithmetic.LongDelegate: **matches(Object, Object)** - Method in class org.jdesktop.el.impl.lang.ELArithmetic: **matches(String, String)** - Method in class org.jdesktop.el.impl.lang.FunctionMapperImpl.Function: **Math()** - Method in class org.jdesktop.el.impl.parser.ELParser: **maxNextCharInd** - Variable in class org.jdesktop.el.impl.parser.SimpleCharStream: **memento** - Variable in class org.jdesktop.el.impl.lang.FunctionMapperFactory: **MessageFactory** - Class in org.jdesktop.el.impl.util: **MessageFactory()** - Constructor for class org.jdesktop.el.impl.util.MessageFactory: **MethodExpression** - Class in org.jdesktop.el: An `Expression` that refers to a method on an object. **MethodExpression()** - Constructor for class org.jdesktop.el.MethodExpression: **MethodExpressionImpl** - Class in org.jdesktop.el.impl: An `Expression` that refers to a method on an object. **MethodExpressionImpl()** - Constructor for class org.jdesktop.el.impl.MethodExpressionImpl: **MethodExpressionImpl(String, Node, FunctionMapper, VariableMapper, Class, Class[])** - Constructor for class org.jdesktop.el.impl.MethodExpressionImpl: **MethodExpressionLiteral** - Class in org.jdesktop.el.impl: **MethodExpressionLiteral()** - Constructor for class org.jdesktop.el.impl.MethodExpressionLiteral: **MethodExpressionLiteral(String, Class, Class[])** - Constructor for class org.jdesktop.el.impl.MethodExpressionLiteral: **MethodInfo** - Class in org.jdesktop.el: Holds information about a method that a `MethodExpression` evaluated to. **MethodInfo(String, Class<?>, Class<?>[])** - Constructor for class org.jdesktop.el.MethodInfo: Creates a new instance of `MethodInfo` with the given information. **MethodNotFoundException** - Exception in org.jdesktop.el: Thrown when a method could not be found while evaluating a `MethodExpression`. **MethodNotFoundException()** - Constructor for exception org.jdesktop.el.MethodNotFoundException: Creates a `MethodNotFoundException` with no detail message. **MethodNotFoundException(String)** - Constructor for exception org.jdesktop.el.MethodNotFoundException: Creates a `MethodNotFoundException` with the provided detail message. **MethodNotFoundException(Throwable)** - Constructor for exception org.jdesktop.el.MethodNotFoundException: Creates a `MethodNotFoundException` with the given root cause. **MethodNotFoundException(String, Throwable)** - Constructor for exception org.jdesktop.el.MethodNotFoundException: Creates a `MethodNotFoundException` with the given detail message and root cause. **MINUS** - Static variable in interface org.jdesktop.el.impl.parser.ELParserConstants: **mod(Number, Number)** - Method in class org.jdesktop.el.impl.lang.ELArithmetic.BigDecimalDelegate: **mod(Number, Number)** - Method in class org.jdesktop.el.impl.lang.ELArithmetic.BigIntegerDelegate: **mod(Number, Number)** - Method in class org.jdesktop.el.impl.lang.ELArithmetic.DoubleDelegate: **mod(Number, Number)** - Method in class org.jdesktop.el.impl.lang.ELArithmetic.LongDelegate: **mod(Object, Object)** - Static method in class org.jdesktop.el.impl.lang.ELArithmetic: **mod(Number, Number)** - Method in class org.jdesktop.el.impl.lang.ELArithmetic: **MOD0** - Static variable in interface org.jdesktop.el.impl.parser.ELParserConstants: **MOD1** - Static variable in interface org.jdesktop.el.impl.parser.ELParserConstants: **MULT** - Static variable in interface org.jdesktop.el.impl.parser.ELParserConstants: **Multiplication()** - Method in class org.jdesktop.el.impl.parser.ELParser: **multiply(Number, Number)** - Method in class org.jdesktop.el.impl.lang.ELArithmetic.BigDecimalDelegate: **multiply(Number, Number)** - Method in class org.jdesktop.el.impl.lang.ELArithmetic.BigIntegerDelegate: **multiply(Number, Number)** - Method in class org.jdesktop.el.impl.lang.ELArithmetic.DoubleDelegate: **multiply(Number, Number)** - Method in class org.jdesktop.el.impl.lang.ELArithmetic.LongDelegate: **multiply(Object, Object)** - Static method in class org.jdesktop.el.impl.lang.ELArithmetic: **multiply(Number, Number)** - Method in class org.jdesktop.el.impl.lang.ELArithmetic

---


|  |  |  |
| --- | --- | --- |
| **Overview** \| Package \| Class \| Use \| **Tree** \| **Deprecated** \| **Index** \| **Help** | |  |
| **PREV LETTER**   **NEXT LETTER** | **FRAMES**    **NO FRAMES**     **All Classes** |  |


A B C D E F G H I J K L M N O P Q R S T U V W 

---
